# Histological Evaluation of Subcutaneous Tissue Reactions to a Novel Bilayer Polycaprolactone/Silk Fibroin/Strontium Carbonate Nanofibrous Membrane for Guided Bone Regeneration: A Study in Rabbits

**DOI:** 10.1002/cre2.70140

**Published:** 2025-04-30

**Authors:** Lida Kheiri, Arash Golestaneh, Mehdi Mehdikhani, Sayed Mohammad Razavi, Niloofar Etemadi

**Affiliations:** ^1^ Department of Oral and Maxillofacial Surgery School of Dentistry, Islamic Azad University, Isfahan (Khorasgan) Branch Isfahan Iran; ^2^ Dental Research Center, Dental Research Institute, School of Dentistry Isfahan University of Medical Sciences Isfahan Iran; ^3^ Department of Biomedical Engineering, Faculty of Engineering University of Isfahan Isfahan Iran; ^4^ Department of Oral and Maxillofacial Pathology, Dental Implants Research Center School of Dentistry, Isfahan University of Medical Sciences Isfahan Iran; ^5^ Department of Materials Engineering, Najafabad Branch Islamic Azad University Najafabad Iran; ^6^ Department of Biophysics, Institute of Quantum Biophysics Sungkyunkwan University Suwon Republic of Korea

**Keywords:** bilayer membrane, polycaprolactone, silk fibroin, strontium carbonate

## Abstract

**Objectives:**

The selection of appropriate biomaterial for guided bone regeneration is challenging. The blending of polymers is a simple method to retain their characteristics and to compensate for the drawbacks of each component. The release of Sr^+2^ (strontium) ions from the polycaprolactone/strontium carbonate (PCL/SrC) nanocomposite is the main reason of enhanced osteogenesis. The most important reasons of clinical failure after using biomaterials include infections and lack of tissue‐integration. Modifications of silk fibroin (SF)–based membranes improved new bone formation in animal studies without inflammatory reaction. The aim of the present study was to compare biological response of the subcutaneous connective tissue to a novel bilayer PCL (60 wt%)/SF (20 wt%)/SrC (20 wt%) membrane to a commercially available collagenous membrane.

**Material and Methods:**

Eighteen male New Zealand rabbits were randomly divided into three groups, and all received subcutaneously the following materials: novel bilayer membrane, commercial membrane, and empty defect as control group, which were tested after 7, 14, and 28 days. The type and severity of inflammation, granulation tissue, and fibrous tissue were assessed.

**Results:**

The connective tissue surrounding the implanted samples of each group exhibited the presence of similar cells close to the control groups. Statistical analyses showed no significant differences between the specimens in each time period.

**Conclusions:**

In general, the novel bilayer nanocomposite membrane was a biocompatible material and produces a similar subcutaneous response compared to commercially available membrane. Besides, it demonstrated promise for guided bone regeneration technique for treating the osseous defects of oral and maxillofacial region.

## 1 Introduction

Although bone is capable of self‐regeneration, reconstruction of large bone defects is a complex biological process that has been challenging for clinicians (Wang et al. 2020; Luz et al. 2020; Khojasteh et al. 2017; da Costa Pereira et al. 2019; Du et al. 2019; Kim et al. 2014; Moe et al. 2021). Guided bone regeneration (GBR) is known as an effective method for bone reconstruction compared to using bone grafts alone by applying membranes that mechanically prevent non‐osteogenic cells migration from soft tissues (Khojasteh et al. 2017; da Costa Pereira et al. 2019; Soltani Dehnavi et al. 2018; Haghighat et al. 2019; Lu et al. 2015; Masoudi Rad et al. 2017; Song et al. 2011). Among resorbable membranes, collagen is the most commonly used as it performs multiple roles including promoting hemostasis by attracting platelets and increasing fibrin linkages, supporting osteogenesis by providing a surface for attachment of bone‐forming cells, acting as a scaffold for the development of neovascularization, and increasing fibroblasts chemotaxis (Ramalingam et al. 2016). The shortcomings of collagen are expensiveness and the risk of disease transmission, rapid degradation, and insufficient mechanical strength that might collapses in wet in spite of excellent biocompatibility and cell affinity (Wang et al. 2020; da Costa Pereira et al. 2019; Soltani Dehnavi et al. 2018; Haghighat et al. 2019; Lu et al. 2015; Song et al. 2011; Aldemir Dikici et al. 2019; Korzinskas et al. 2018; Kim et al. 2005; Pripatnanont et al. 2021). To overcome the limitations of natural membranes, synthetic biomaterials such as polymers and composites have become increasingly popular due to their biocompatibility and ease of manipulation (Wang et al. 2020; Haghighat et al. 2019). Numerous studies have explored the potential of degradable synthetic materials for developing suitable membranes, which may provide improved mechanical strength and stiffness, leading to better maintenance of space and protection of the surgical site (Kim et al. 2005; Cai et al. 2017). Few papers focus on multilayer membrane structures similar to natural oral soft tissue (Watcharajittanont et al. 2019).

Although polycaprolactone (PCL)—an inert, semi‐crystalline aliphatic polymer with outstanding compatibility, excellent solubility, easy handling, sterilization capability, inexpensive manufacturing process, and controlled degradation—has been successfully used in bone tissue engineering, it imposes limitations due to low stiffness and a hydrophobic nature that restricts biomaterial/cell interaction (Soltani Dehnavi et al. 2018; Aldemir Dikici et al. 2019; Shaltooki et al. 2019; Terzopoulou et al. 2019; Nazeer et al. 2019). Furthermore, PCL does not release acidic degradation products that could affect cell growth (Terzopoulou et al. 2019). To improve its properties and osteoconductivity, a composite approach in which polymers are combined with bioactive materials as silk fibroin (SF) might be helpful (Soltani Dehnavi et al. 2018; Shaltooki et al. 2019; Wang et al. 2016). SF is a natural polymer, collagen‐like fiber with suitable mechanical strength, toughness, biocompatibility, morphologic flexibility, biodegradability, permeability, anti‐thrombogenicity, low cost, and noninflammatory characteristics, which not only regulates mineralization but also improves the osteoconductivity (Wang et al. 2020; Du et al. 2019; Kim et al. 2014; Moe et al. 2021; Lu et al. 2015; Song et al. 2011; Kim et al. 2005; Pripatnanont et al. 2021; Cai et al. 2017; Watcharajittanont et al. 2019). Similarly, silk proteins improve alkaline phosphatase (ALP) activity and osteogenic differentiation of osteoblasts plus proliferation and adhesion of mesenchymal stem cells (MSCs) (Wang et al. 2020). Incorporation of other substances such as strontium chondroitin sulfate can modify brittle SF membranes to be suitable for GBR (Kim et al. 2014; Pripatnanont et al. 2021; Li et al. 2021). Strontium (Sr) plays a vital role in bone mineralization due to its similarity to calcium (Ca) (in relation to charge, size, and cellular transport pathway), stimulates osteoblasts and incites osteogenesis, while decreasing bone resorption, through the inhibition of osteoclasts (Wang et al. 2020; Luz et al. 2020; Aldemir Dikici et al. 2019; Shaltooki et al. 2019; Terzopoulou et al. 2019; Meka et al. 2016). It has been shown that 0%–100% of Ca ions substituted with Sr ions and its anabolic influence and bone regenerative properties were confirmed (Shaltooki et al. 2019). The release of Sr^+2^ ions from the PCL/SrC composite were the main reason of enhanced osteogenesis compared to the pure PCL nanofibrous scaffolds (Meka et al. 2016).

The most important reasons of clinical failure after using biomaterials include infections and lack of tissue integration (Mehdikhani‐Nahrkhalaji et al. 2011; Xue et al. 2014). The first step in assessing the biological response to implanted materials is their biocompatibility to host tissues, especially subcutaneous tissue in comparison to a control material (Korzinskas et al. 2018; Muñoz et al. 2019). Thus, the understanding of the foreign body reaction, and interactions of the immune system with a biomaterial is necessary to ensure its safety, biocompatibility, and functionality (Korzinskas et al. 2018).

A study was performed to fabricate a novel, bioactive, and biodegradable nanocomposite bilayer membrane based on PCL, which demonstrated that the bilayer structure doubled the optimum mechanical properties and the addition of SrCO_3_ up to 15%–20% increased ALP activity, Ca deposition, and bioactivity (Terzopoulou et al. 2019; Etemadi et al. 2021). Therefore, the aim of this study was to compare the efficacy of the subcutaneous connective tissue reactions to a previously fabricated and in vitro‐evaluated novel bilayer PCL (60 wt%)/SF (20 wt%)/Sr (20 wt%) membrane (Etemadi et al. 2021) to a commercially available collagenous membrane to determine inflammation levels and biological response.

## 2 Materials and Methods

### 2.1 Membrane Fabrication

**Figure 1:**
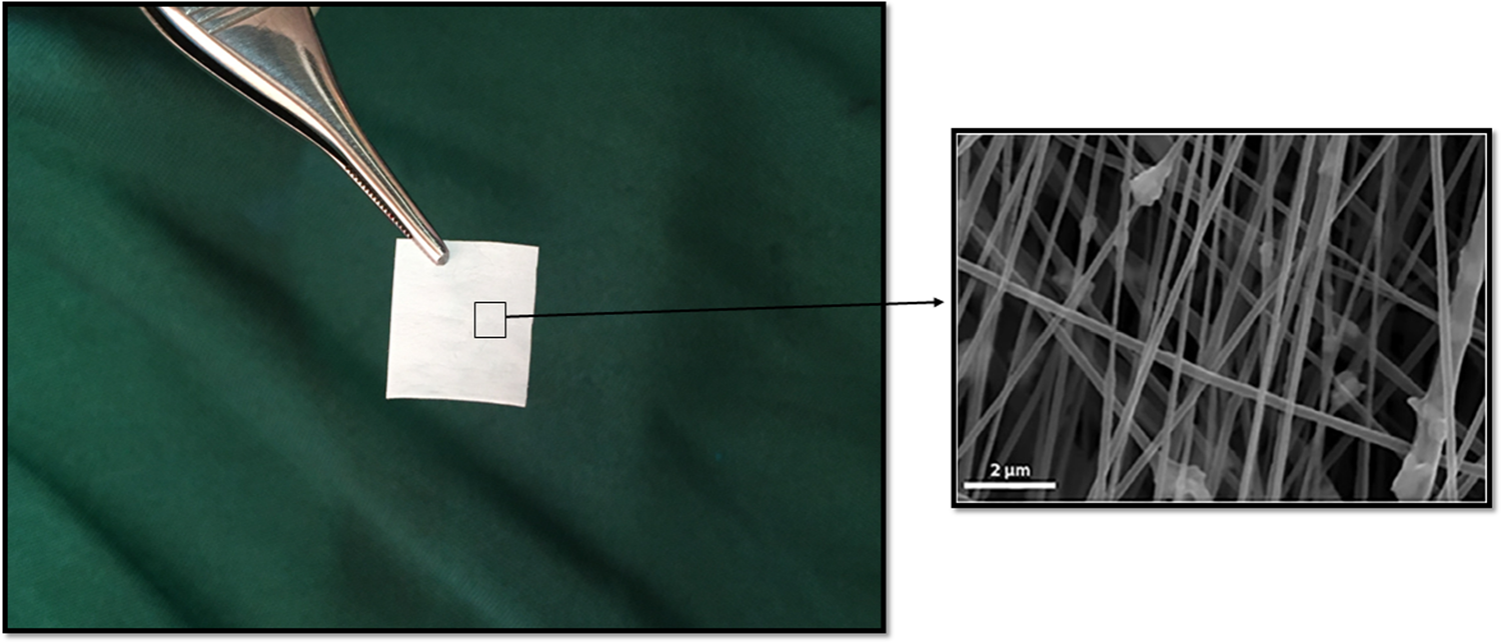
(A) The selected sample of bilayer PCL/SF/SrC membrane (Etemadi et al. 2021). (B) SEM micrograph of the novel bilayer membrane with random nanofibrous structure and the presence of the Sr/C nanoparticles (Etemadi et al. 2021).

Optimum nanofibrous bilayer membrane was fabricated based on the technique proposed in the previous study and nanofibrous bilayer membrane containing 20 wt% SrCO_3_, 20 wt% SF, and 60 wt% PCL was chosen as an optimum sample to use in the present animal study (Figure 1) (Etemadi et al. 2021).

### 2.2 Experimental Design

The current study for animal experimentation was approved by the institutional Animal Use Ethics Committee of Islamic Azad University (the protocol no. is IR.IAU.KHUISF.REC.1400.185), and the guidelines for the care and use of laboratory animals were followed. Experiments were reported according to the ARRIVE guidelines for relevant items (Percie du Sert et al. 2020), and the study was carried out in accordance with the UK Animals (Scientific Procedures) Act, 1986 and associated guidelines, EU Directive 2010/63/EU for animal experiments.

### 2.3 Animal Model

**Figure 2:**
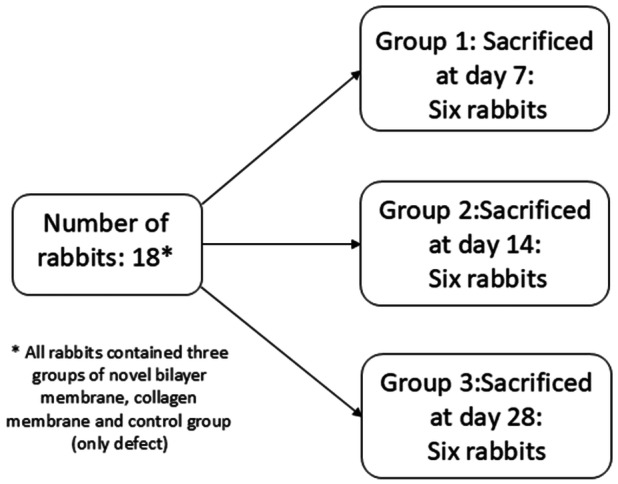
Study groups of rabbits in the study.

Eighteen male New Zealand rabbits with a mean weight of 2.5–3 kg were used. A veterinarian examined the rabbits and confirmed their health. Two weeks before surgery, the rabbits were placed in separate cages under standard laboratory conditions and were fed a standard diet, pelleted feed (Nuvilab CR1, Nuvital, Brazil) and water ad libitum. Animals were randomly divided into three groups of six and all received the following materials: novel bilayer membrane, collagen membrane, and empty defect as control (Figure 2).

### 2.4 Surgical Procedure

**Figure 3:**
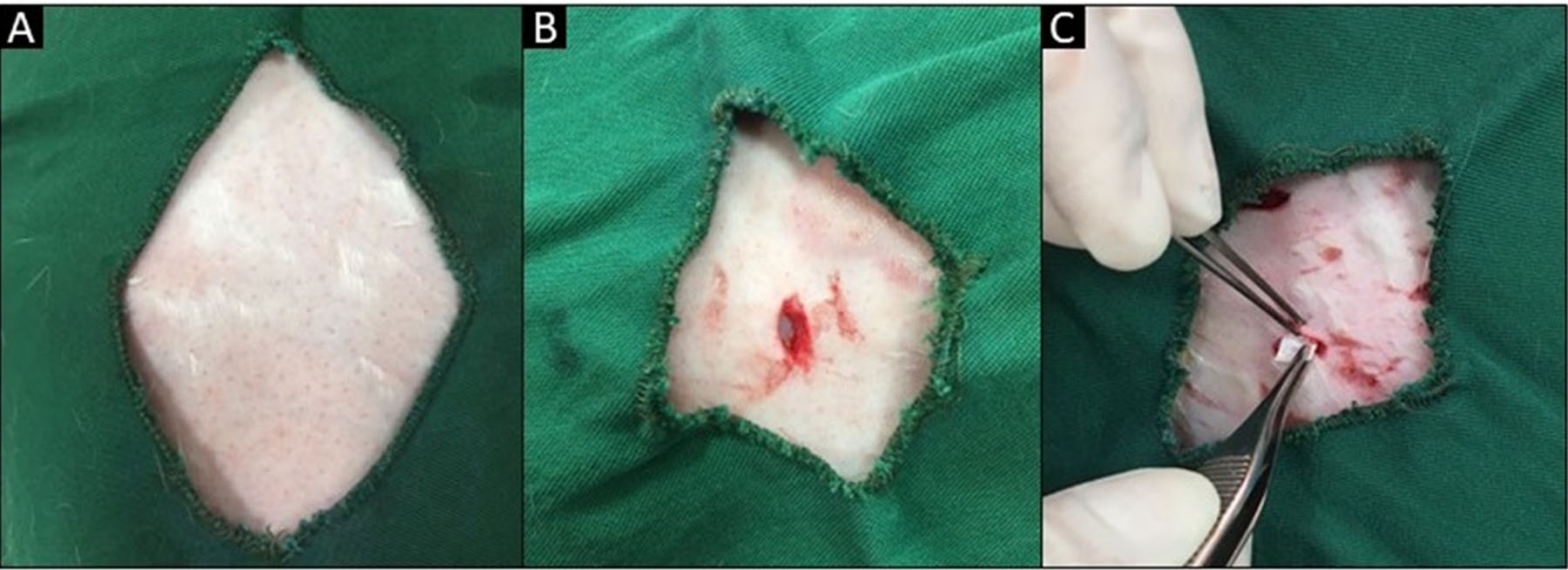
Surgical procedure. (A) Shaved surgical region. (B) Dermal pocket was prepared. (C) Subcutaneous membrane placement.

After a 24‐h fast, surgery was performed under aseptic conditions and general anesthesia induced by intramuscular injection of ketamine, 60 mg/kg (Yuhan Co., Korea) and 5 mg/kg xylazine (Rompun; Bayer HealthCare, Leverkusen, Germany), respectively. The surgical regions were shaved and disinfected with povidone‐iodine, followed by local anesthesia induced by 2% lidocaine HCl/epinephrine (Xylopen; 20 mg/12.5 mg/mL [1.8 mL]; Exir Pharmaceutical Co, Borujerd, Iran). The place of implantation was surrounded by sterile gauzes. Three dermal pockets were made by 1‐cm‐long surgical incision using no. 15 blade (Romed, Holland) in the left side of the skin in the epithelium of dorsal thoracic zone in a head‐to‐tail direction, 1 cm lateral to the vertebral column, followed by divulsion of the muscular fascia skin with the aid of scalpel and blunt‐tipped scissors to expose the subcutaneous tissue for insertion of the membrane. To prevent interactions of materials, membranes were placed at least 2 cm away from each other. Every membrane measuring 1 × 1 cm was placed in each pocket, and the skin was closed with 3/0 nylon sutures (Ethicon Inc., United States) (Figure 3) (Mehdikhani‐Nahrkhalaji et al. 2011).

### 2.5 Postoperative Care

In the postoperative period, the animals were kept in the Animal Experimentation Laboratory (AEL/UFF), divided in isolators based on their experimental groups, and received food and water ad libitum. All animals received penicillin 10,000 U/day immediately after implantation and continued for 3 days. Meloxicam (5 mg/kg; Eurofarma Laboratorios LTDA, São Paulo, SP, Brazil) was administered subcutaneously on the day of surgery and for 2 days thereafter.

### 2.6 Histopathology Analysis

Histopathological analysis was performed to demonstrate tissue reaction to the implanted nanofibrous membrane. At 7, 14, and 28 days after surgical implantation, animals were sacrificed by administering high doses of anesthetics, the dorsal skin was shaved, and a piece of cutaneous tissue with the connective tissue around them was taken (1 cm^3^ ± 5 mm safety margin) from each implantation site and was fixed in 10% formalin solution (37% formaldehyde; Merck, Germany). Samples underwent a dehydration in serial alcohol dilutions (ethyl alcohol and xylol series) and paraffination process to make paraffin blocks and were cut into 5–6 μm thick slices by a microtome, which stained with Hematoxylin and Eosin (H&E). Histological evaluations were made under a light microscope at ×100 and ×400 magnifications (Nikon Eclipse E400, Tokyo, Japan). These images were captured using a high‐resolution digital camera (Sony HD DSC HX9V 16.2 Mega Pixels, Tokyo, Japan). The cells of connective tissue and cells of inflammatory response were counted. The pathologist was blind to the procedure. Inflammatory processes, including the presence of acute‐phase cells (neutrophils and polymorphonuclear cells [PMN]) and chronic‐phase cells (lymphocytes and foreign body type‐multinucleated giant cells) were evaluated. The extent of inflammatory reactions was graded by counting the average number of inflammatory cells in 10 fields: None (0): scattered chronic inflammatory cell; mild (1): infiltration of inflammatory cells, wavy collagen fiber deposits, and fibrosis; moderate (2): dense infiltration of inflammatory cells plus limited areas of tissue edema and vascular congestion; and, finally, severe (3): very dense infiltration of acute and chronic inflammatory cells plus widespread edematous areas and vascular congestion along with fibrin deposits. A high infiltrating cell density was taken as an indication of poor biocompatibility (Mehdikhani‐Nahrkhalaji et al. 2011).

### 2.7 Statistical Analysis

The sample size to compare the mean number of highly and chronically inflamed cells between three groups for a two‐sided test at a significant level of 5% (*α* = 0.05), with a test power of 80% (*β* = 0.2) and to detect a minimum difference of at least 70% greater than the standard deviation (*δ* = 1.7σ), was calculated according to the following formula to be equal to six subjects in each experimental group. Statistical analysis was performed using Kruskal–Wallis analysis and Fischer tests. The value of *p* < 0.05 was used to indicate statistically significant differences. Calculations were done using the SPSS 24.

## 3 Results

### 3.1 Gross Findings

All animals recovered from the surgery, remained in good health, and their appearance and physical activity were normal. All wounds healed well without significant infection or complications, and no significant weight reduction and rejection of the membranes were noted. During the extraction surgery, the color and tissue structure were normal, and no lesions were noted. Bilayer membranes began to exhibit fragmentation with tissue invasion, which was evident at the removal surgery. Most control membranes did not adhere to the subcutaneous tissue macroscopically; therefore, during the removal of the samples, they were detached from the tissue. All membranes sustained shape stability with no collapse or breakage. Meanwhile, the thicknesses of the membranes decrease gradually, indicating that the membranes degraded from the surface, which is good to perform the barrier function performance.

### 3.2 Histological Findings

**Figure 4:**
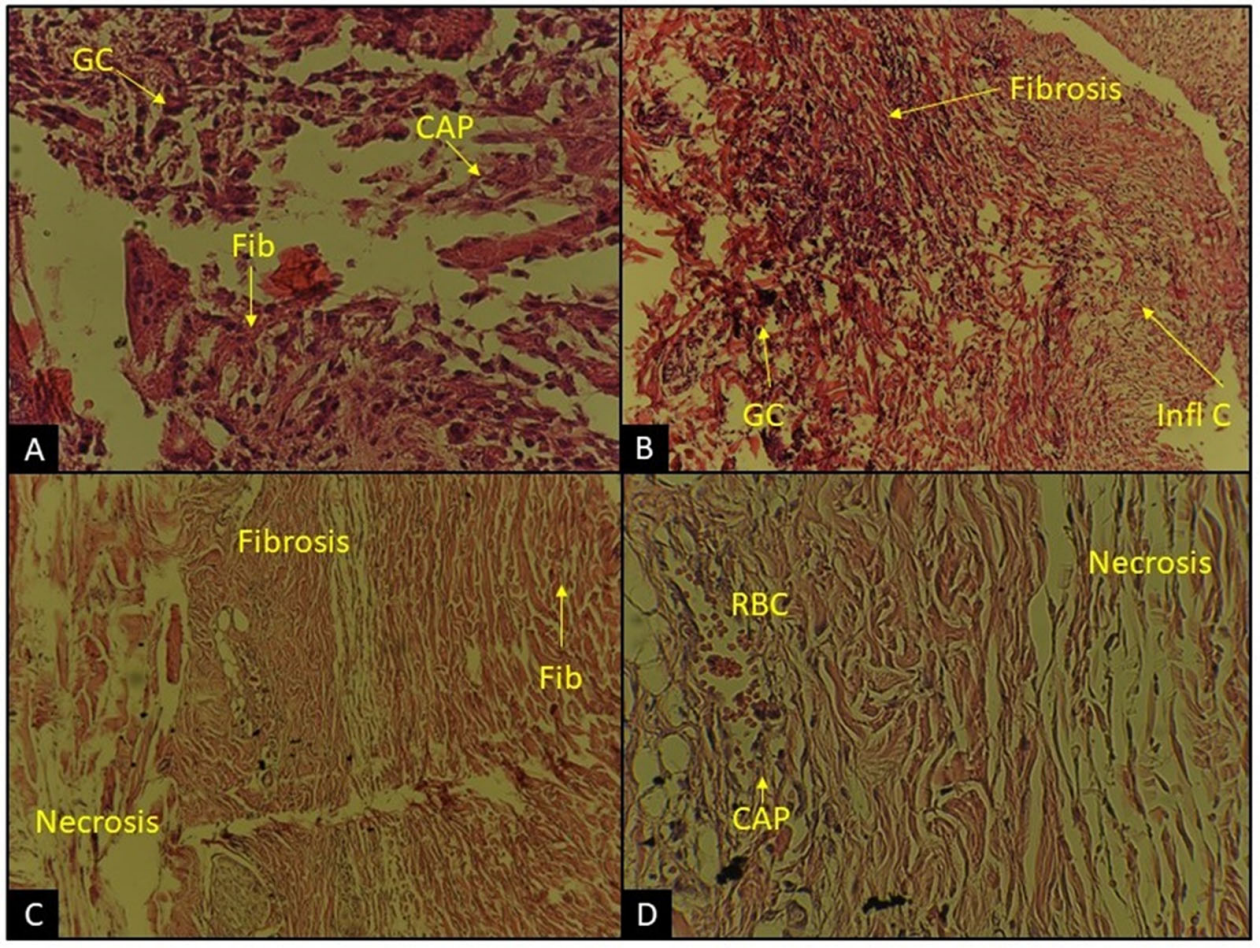
Subcutaneous tissue reaction histological images (H&E stain). (A) Bilayer membrane at day 28 (×400). (B) Control membrane at day 7 (×100). (C) Bilayer membrane at day 7 (×100). (D) Bilayer membrane at day 7 (×400). CAP: capillary; Fib: fibroblast; GC: giant cell; Infl C: inflammatory cell; RBC: red blood cell.

The descriptive analysis of the tissue response to the membranes was evaluated in the different groups: the presence of inflammatory cells, fibrosis, necrosis, giant cells, and granulation tissue. The connective tissue surrounding the implanted samples of each examined group displayed the presence of similar cells. The number of connective tissue cells was almost similar for all materials, close to the control group (Figure 4).

### 3.3 Connective Tissue

#### 3.3.1 Fibrosis

Fibrosis was not significantly different at days 7, 14, and 28 post‐implantations within and between groups. Furthermore, after 7 days, fibrosis score in synthetic membrane samples was the lowest in comparison with collagen and control groups that the highest was collagen membrane. Fibrosis tissue was the lowest in collagen group. Surprisingly, collagen membrane on day 28 showed a higher fibrosis score compared to others. Outcomes did not show significant differences between groups in three periods of follow‐up, which demonstrates acceptable overall biocompatibility.

### 3.4 Granulation Tissue

There was a persistence of granulation tissue. The granulation tissue layer was thicker for collagen membrane on day 7 and for bilayer membrane on day 14. However, at day 28, the thickness of the granulation tissue decreased to a thin layer for new membrane group than the other two groups. There were no significant differences between groups on 7, 14, and 28 days.

### 3.5 Type and Intensity of Inflammation

The extent of inflammation was manually assessed and quantified based on the number and distribution of inflammatory cells. An inflammatory tissue reaction was observed within the implantation beds of groups after 7, 14, and 28 days postimplantation (Figure 4). The H&E staining results observed in optical microscope images are illustrated in Figure 4. The inflammatory reactions were considerable on day 7, whereas they reduced on days 14 and 28. Inflammatory reaction between groups at 7, 14, and 28 days was not significant (*p* > 0.05) (Table 1). The formation of thinner inflammatory fibrous capsules is taken as an indication of poorer biocompatibility.

**Table 1:** Frequency distribution of inflammation type (percentage) between bilayer membrane, collagen membrane, and control groups after 7, 14, and 28 days.

Assessment period	Inflammation type	Bilayer membrane	Collagen membrane	Control group	*p* value
Day 7	Acute	50.0	50.0	50.0	1.00
	Chronic	50.0	50.0	50.0	
Day 14	Acute	83.3	100	83.3	1.00
	Chronic	16.7	0.0	16.7	
Day 28	Acute	100	66.7	83.3	0.735
	Chronic	0.0	33.3	16.7	
*p* value		0.481	0.207	0.230	

### 3.6 Necrosis

In addition, the degree of degeneration (debris) was determined by morphological alterations due to necrosis extension. Necrosis was observed in 7‐day (bilayer membrane and empty area samples) group and 28‐day group (both for bilayer membrane samples).

### 3.7 Giant Cell

Giant cells have been observed in all three groups, especially after 7 days.

## 4 Discussion

In summary, both materials were considered biocompatible and promoted decreases in the inflammatory response and increases in connective tissue repair over the experimental times. The most functional stability of the tested material and low biodegradation during the experimental period were demonstrated.

**Membrane Features:** Not only should ideal GBR membranes promote bone regeneration, they should also be able to protect the wound site from ingrowth of soft tissue, without creating infection and inflammation (Luz et al. 2020; da Costa Pereira et al. 2019; Haghighat et al. 2019; Lu et al. 2015). Although appropriate mechanical and bioactive properties are required for the membranes, biodegradability rate is also important to avoid the second surgery (Haghighat et al. 2019; Masoudi Rad et al. 2017). Designing a membrane to perform both functions is a challenge for materials scientists and surgeons (Moe et al. 2021). The fabricated bilayer PCL/SF/SrC membrane not only introduced all appropriate physical and chemical properties but also demonstrated promising potential for oral and maxillofacial repair and regeneration in vitro (Etemadi et al. 2021). The study by Shim et al. revealed that PCL and PCL/TCP membranes could be more easily surgically manipulated compared to commercial collagen membranes (Shim et al. 2017). The novel membrane was manipulated easily during surgery, which is one of the most fundamental features of GBR membranes. Mechanical resilience when exposed to wet conditions must allow for ease of manipulation in surgeries (Haghighat et al. 2019).

**Histological Analysis:** The tissue response to biomaterials is a cascade that primarily involves macrophages as key elements expressing both pro‐ and anti‐inflammatory molecules (Korzinskas et al. 2018). The presence of neutrophils at the graft site, and generally from 48 h to almost a week, indicates acute inflammation (Muñoz et al. 2019). PMN cells enable optimal healing and tissue regeneration (Muñoz et al. 2019). The normal acute‐phase inflammatory process is characterized by vasodilation, protein adsorption onto the biomaterial surface, and the presence of high‐density leukocytes (neutrophils) (Cai et al. 2017). In contrast, the chronic‐phase, or initial phase, of tissue regeneration is identified by the presence of lymphocytes, macrophages, mesenchymal cells, and fibroblasts, where the fibroblasts deposit the collagen matrix and encapsulate the biomaterial in a fibrous tissue layer (Cai et al. 2017). The results presented in this study are consistent with those of other authors and substantiated by the presence of early inflammation at all assessed implantation sites. This could be due to the surgical procedure and did not differ from the pattern in the control group. Additionally, the protective effects on the immune system may cause inflammation to last longer than expected, but should resolve over time (Muñoz et al. 2019). Similar cells were observed around all materials that are typical for a biological organism response to the introduced implant including lymphocytes, neutrophils, macrophages, and sometimes even giant cells and strong inflammation as the results observed by researchers testing the commonly considered as biocompatible PCL/PLLA copolymers (Zair et al. 2019). No remarkable immunologic rejection reaction was noted after implantation of membranes. Kim et al. applied electrospun SF for GBR application and showed that this membrane had good biocompatibility without evidence of inflammatory response (Kim et al. 2005; Atrian et al. 2019). A good healing response and improved biocompatibility may be due to granulation tissue formation and regeneration (Muñoz et al. 2019). It has to be remembered that physiological levels of inflammation are present under healthy conditions, as the initial response of M1 macrophages is a process required to result in high levels of pro‐inflammatory cytokine expression on biomaterials (Korzinskas et al. 2018). Sustained pro‐inflammatory response is associated with material damages, as it will induce a severe foreign body reaction or fibrous encapsulation (Korzinskas et al. 2018). These results suggest that the novel bilayer membrane induces an inflammatory tissue response comparable to the collagen membrane, which is believed to be biocompatible. Immunohistochemistry does not allow description of the expression levels of various cytokines or mediators by macrophages involved in the inflammatory response, so the results of this study provide limited information on the extent of the inflammatory response. This leads to the conclusion that biocompatibility analysis may also require standardized in vitro test systems that include cell types involved in foreign body responses to biomaterials. Granulation tissue thickness is inversely proportional to how well the implanted material is tolerated by the host tissue (Zhang et al. 2010). The inflammatory response was reduced during experimental periods. In vivo examinations of the implanted SF nanofibrous membranes showed a negligible inflammatory response including occasional lymphocytes, eosinophils, foreign‐body giant cells, and the subsequent formation of a relatively thick fibrous capsule (Kim et al. 2005).

**Biodegradation:** Phagocytic activity in the first days after surgery determines membrane biodegradation, which has a complex mechanism (Muñoz et al. 2019). Degradation typically occurs in four stages: hydration, strength loss, mass integrity loss, and cell phagocytosis. As biodegradable materials are typically grafted under hemorrhagic conditions, which are hydrophilic, differences in the water solubility state of membranes can affect the degree of hydration, resulting in loss of strength and mass integrity (Kim et al. 2014; Song et al. 2011). Host factors, such as the treatment site and the host immune system and also surgical skills and related medical conditions, can also affect degradation. Various factors can influence the rate of degradation, which is useful for understanding the different bone growth patterns of each membrane (Kim et al. 2014; Song et al. 2011). For beneficial clinical results, the GBR membranes should remain in the surgical site for up to 6 months (Haghighat et al. 2019). Haghighat et al. reported that PCL and PCL/TCP membranes tolerated the physiological environment for 8 weeks after implantation, which is a disadvantage for successful clinical outcome (Haghighat et al. 2019). The present results demonstrate that most membranes were intact after 7 days of implantation, but after 28 days, they were partially fragmented and had lower mechanical stability in comparison with 14 days post implantation. It is verified that both membranes are biocompatible with the subcutaneous connective tissue of rabbit. However, our study failed to identify clear differences in degradation rate or relationship with bone regeneration between the two membrane treatment groups. In rat subcutaneous connective tissue, the degradation of the membranes (SF, PLA, and collagen) had started at 8 weeks, and degraded fragments of the membranes were lined up around the membranes at 12 weeks. Another study demonstrated that silk, as a protein, is susceptible to proteolytic degradation in vivo and over longer time periods in vivo will slowly be absorbed (Kim et al. 2005). The study suggests that the silk provided the mechanical strength and space necessary for guided bone formation (Kim et al. 2005). In that study, SF after removal of sericin was used to reduce inflammation and to promote biocompatibility (Kim et al. 2005). To play the role as a barrier, absorbable membranes should remain for at least 3–4 weeks (da Costa Pereira et al. 2019). Degradation time may accompany the time required for new bone formation. Foreign body or Langhans multinucleated giant cells are actively involved in the resorption process over the long term. Cai et al. reported that SF membrane provides a secluded space to prevent over proliferation and downgrowth of the epithelium and fibroblasts from normal tissue healing (Cai et al. 2017). The incorporation of Sr^2+^ ions induces significant bioactivity, which is further enhanced in the presence of ibandronate (Terzopoulou et al. 2019).

**Biocompatibility:** According to Bell and Beirne, the fibrosis in the implant‐tissue interphase is a biocompatibility criterion, and the presence of mature connective tissue indicates a successful integration with the host tissues (Bell and Beirne 1988). Fibrin acts as the main scaffold for collagen and clot formation and leads to adequate levels of fibronectin, responsible for endothelial platelet adhesion (Muñoz et al. 2019). Some authors suggest that fibrosis along with other pathobiological processes such as acute inflammation and granulation tissue formation are common features after material implantation and help improve healing and biological degradation of membrane (Muñoz et al. 2019). In this study, we show that fibrosis was generally highest after 7 days and lowest at 28 days while being almost similar between groups. These findings are in accordance with other authors that describe the presence of mature connective tissue in the material‐tissue interphase as an indicator of good implantation and healing response. Although synthetic membrane fibrosis was higher on day 7 postimplantation compared to control groups, resorption is expected later; hence, longer time‐point studies are strongly suggested (Muñoz et al. 2019).

## 5 Conclusion

Results proved to a large extent that the novel bilayer membrane was biocompatible and produces a similar subcutaneous response compared to commercially available membrane. Thereafter, it may be concluded that this combination was well tolerated by the tissues and was a promising membrane for GBR method in oral and maxillofacial region. Further study on in vivo barrier efficacy of bone formation is still needed to determine if these membranes can be introduced to clinical practice in the future.

## Author Contributions

**Lida Kheiri:** investigation, methodology, visualization, writing – original draft preparation. **Arash Golestaneh:** supervision. **Mehdi Mehdikhani:** conceptualization, supervision, validation. **Sayed Mohammad Razavi:** methodology, project administration, writing – review and editing. **Niloofar Etemadi:** resources.

## Conflicts of Interest

The authors declare no conflicts of interest.

## Data Availability

The authors have nothing to report.
